# Insights from *Melipona bicolor* hybrid genome assembly: a stingless bee genome with chromosome-level scaffold

**DOI:** 10.1186/s12864-024-10075-x

**Published:** 2024-02-13

**Authors:** Natalia de Souza Araujo, Fernando Ogihara, Pedro Mariano Martins, Maria Cristina Arias

**Affiliations:** 1 Evolutionary Biology & Ecology, Université Libre de Bruxelles – ULB, Brussels, Belgium; 2 Laboratory of Genetics and Evolution of Bees, Bioscience Institute, Universidade de São Paulo – USP, São Paulo, Brazil; 3 Gene Expression and Evolution Laboratory, Bioscience Institute, Universidade de São Paulo – USP, São Paulo, Brazil

**Keywords:** Meliponini, Genome assembly, Venom genes, Eusocial bees, Polygyny

## Abstract

**Background:**

The highly eusocial stingless bees are crucial pollinators of native and agricultural ecosystems. Nevertheless, genomic studies within this bee tribe remain scarce. We present the genome assembly of the stingless bee *Melipona bicolor*. This bee is a remarkable exception to the typical single-queen colony structure, since in this species, multiple queens may coexist and share reproductive duties, resulting in genetically diverse colonies with weak kinship connections. As the only known genuinely polygynous bee, *M. bicolor*’s genome provides a valuable resource for investigating sociality beyond kin selection.

**Results:**

The genome was assembled employing a hybrid approach combining short and long reads, resulting in 241 contigs spanning 259 Mb (N50 of 6.2 Mb and 97.5% complete BUSCOs). Comparative analyses shed light on some evolutionary aspects of stingless bee genomics, including multiple chromosomal rearrangements in *Melipona*. Additionally, we explored the evolution of venom genes in *M. bicolor* and other stingless bees, revealing that, apart from two genes, the conserved repertoire of venom components remains under purifying selection in this clade.

**Conclusion:**

This study advances our understanding of stingless bee genomics, contributing to the conservation efforts of these vital pollinators and offering insights into the evolutionary mechanisms driving their unique adaptations.

**Supplementary Information:**

The online version contains supplementary material available at 10.1186/s12864-024-10075-x.

## Background

The Meliponini, commonly known as the stingless bees, comprise a diverse group of highly eusocial bees that play a crucial role as pollinators of native and crop plants [1–3]. These bees are essential for the sustainable development of local ecosystems, particularly in the Neotropics, as they contribute to the maintenance of native flora diversity while also enhancing agricultural productivity [3, 4]. Nonetheless, the diversity of stingless bees faces constant threats for multiple reasons such as habitat loss, inappropriate beekeeping practices, and the introduction of invasive species [1, 5–7]. Stingless bees also serve as valuable models for behavioral and evolutionary studies given their wide range of adaptations [8] as the tribe comprises about 500 species and 48–61 genera [9, 10]. Despite the importance of the group genomic studies in Meliponini are scarce, only ten stingless bee genomes (excluding the current assembly) have been made available on NCBI until January 2024 [11], and only a couple of these relied on long-read sequencing data. In an effort to diminish this knowledge gap, we performed the genome sequencing, assembly, and annotation of the stingless bee *Melipona bicolor* (Lepeletier, 1836), using a hybrid assembly approach that combined short and long reads data. To gain some insights into stingless bees' genomic evolution, we compared this genome with other corbiculate bee species.

The nuclear genome of *M. bicolor* has the typical chromosome number for *Melipona* species (*n* = 9) [12] and an estimated genome size of 273.84 Mb [13]. Moreover, *M. bicolor* is part of the so-called Group I of *Melipona* species characterized by low heterochromatin content [12, 13]. Like honeybees, almost all stingless bee species exhibit monogynic perennial colonies, where a single reproductive queen resides alongside multiple workers [9]. An intriguing exception to this rule is observed in *Melipona bicolor* [14], an endemic bee species found in the Brazilian Atlantic Rain Forest [10]. In *M. bicolor* colonies, multiple queens may co-exist and share the reproductive duties of the colony [14, 15]. Notably, polygyny is not mandatory in *M. bicolor*, and the number of queens within a colony can fluctuate [15]. Additionally, workers in *M. bicolor* colonies may accept queens from different genetic backgrounds [16], and they may contribute to male production by laying unfertilized eggs [17]. As a result, polygynic colonies of *M. bicolor* exhibit greater genetic diversity and weak kinship connections [15, 16]. This unique characteristic of *M. bicolor* makes it an exceptionally interesting model for investigating the maintenance of sociality beyond kin selection. Future studies based on its genome will be able to further delve into these aspects, providing valuable insights into the complex dynamics of social behavior in this species.

One of the distinguishing features of stingless bees, as their name suggests, is their inability to sting and inject venom because their stinging apparatus is stunted [9]. The sting apparatus is a modified ovipositor found in Hymenoptera, that allows the inoculation of venom into prey and/ or aggressors [18, 19]. The bee venom of the Apini and the Bombini have been extensively characterized, revealing the presence of various substances such as melittin, apamin, hyaluronidase, phospholipase A2, and venom allergens [20–24]. Even though the composition of most venoms is known to be complex and to rapidly vary according to diverse factors such as age, diet, and sex [25, 26], in a comparative study across the Hymenoptera, Koludarov et al. (2022) found that most venom genes are conserved throughout the group. Intriguingly, these authors also reported that, except for the *melittin* gene, stingless bees still have a complete gene repertoire of bee venom components in their genomes. Herein, we incorporate the genome of *M. bicolor* to further investigate the evolutionary trajectory of venom-associated genes within stingless bees.

By expanding our understanding of the genomic landscape of Meliponini, this study contributes to the conservation and sustainable management of these important pollinators. Additionally, it will enable future studies aiming at understanding the evolutionary processes that have shaped the diverse traits and adaptations observed within this important bee group.

## Results

### ***Melipona bicolo****r* genome assembly

We generated 624,925,618 short and paired reads of 100 bp in length and 1,628,680 long subreads with N50 of 13,649 bp. The Falcon assembler, based on long reads only, resulted in an assembly of 249,831,102 bp total size with an N50 of 466,343 bp across 1,048 contigs, while the hybrid assembly with MaSuRCA generated a genome with 277 contigs, of N50 5,245,763 bp and total length of 261,548,481 bp. After merging these two assemblies, scaffolding with transcriptomic data, and polishing we obtained an assembly with 259 contigs, N50 of 6,225,681 bp, and a total length of 260,232,714 bp. From this, we removed one mitochondrial contig and 17 other contigs with low support (i.e., < 1 × of long read coverage) resulting in our final genome assembly with 241 contigs totalizing 259,858,556 bp with an N50 of 6,225,681 bp, L50 of 12, and largest contig size of 36,767,525 bp (Table S1). This final genome assembly and its annotation, as well as all datasets used to generate them, are available at NCBI under the BioProject PRJNA632864, and accession code JAHYIQ01.

**Fig. 1:**
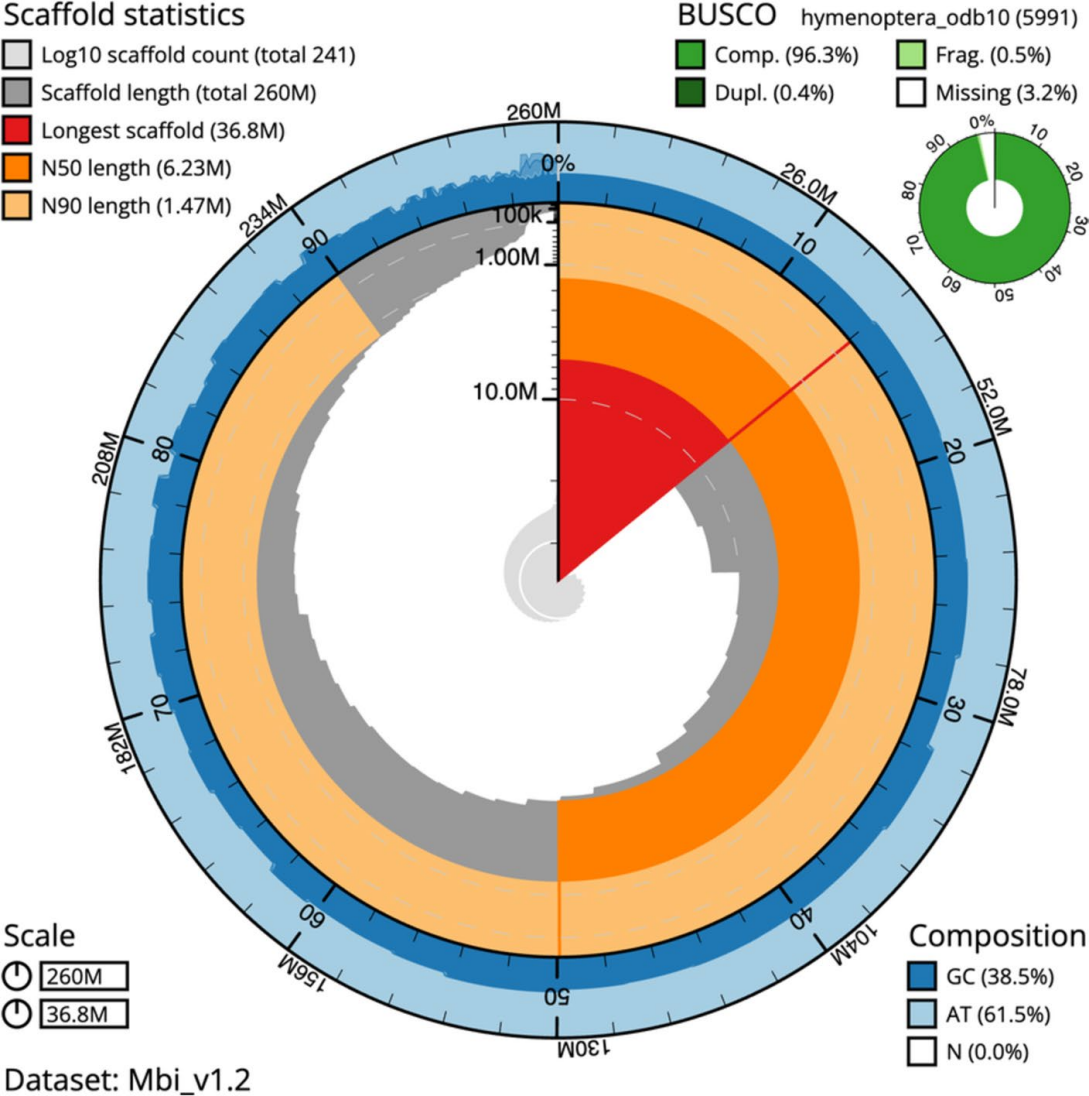
Summary representation of *M. bicolor* genome assembly. The main snailplot is divided into 1,000 size-ordered bins, each representing 0.1% of the total assembly size (259,858,556 bp). Sequence length distribution is shown in dark grey scaled by the largest scaffold of 36,767,525 bp, shown in red). Orange and pale orange indicate the N50 (6,225,681 bp) and N90 (1,474,512 bp), respectively. The pale grey spiral shows the cumulative sequence count on a log scale with white scale lines showing successive orders of magnitude. The blue and pale blue area shows GC and AT distributions. A summary of complete, fragmented, duplicated, and missing BUSCO genes in the hymenoptera_odb10 set is shown in the top right

The mean coverage of long and short reads was respectively 42 (± 15 SD) and 234 (± 626 SD) times. Sequencing coverage was congruent between the two sequencing strategies (Figures S1 and S2). No contaminants were identified through the BlobToolKit analyses as all identified matches to databases were against other bee species (Figure S3). Nevertheless, almost all smaller contigs have a distinct GC proportion when compared to the larger contigs. Small contigs were mostly GC-rich (Fig. 1 and S3), which may indicate that these contigs are most likely composed of repetitive DNA. As seen in Fig. 1, the largest scaffold reached a chromosome size of over 36 Mb for which the long reads were essential to assembly. Notably, keeping contiguity in low GC areas (Figure S1).

**Fig. 2:**
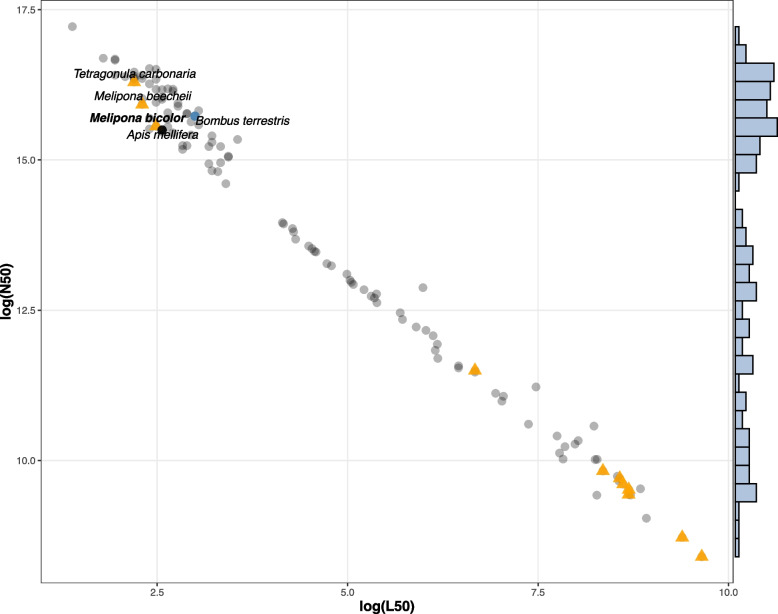
Contigs N50 and L50 values of all bee genomes available at NCBI in January 2024 (120 genomes in total). Values are shown in the log base. Stingless bee genomes (11 genomes) are indicated by orange triangles, the honeybee and the bumblebee genomes are represented by a black and a blue-filled circle, respectively, while all other bee species are represented by grey circles. Only one genome assembly is shown per species, when more than one assembly was available per species the one with the largest scaffold L50 was used. Cumulative frequencies are shown in the steel blue bars

Against the hymenoptera_odb10 (5,991 BUSCO orthologs) the genome of *M. bicolor* contained 96.3% complete BUSCOs (single: 95.9%, duplicated: 0.4%), and 0.5% fragmented and 3.2% missing BUSCO orthologs, respectively. These results are comparable to the best bee genomes available (Table 1), including that of the honeybee and the bumblebee. In terms of contiguity, *M. bicolor* assembly represents a significant improvement from most stingless bee genome assemblies available with a large N50 and reduced contig numbers (Fig. 2). The genomes of the stingless bees *Melipona beecheii* and *Tetragonula carbonaria* are of equivalent quality and also rely on long-read data, but no annotation is available for these species.

**Table 1:** BUSCO comparative analyses of conserved orthology across two high-quality genome assemblies (*B. terrestris* and *A. mellifera*), *M. bicolor* assembly (in bold), and all other stingless bee genomes available at NCBI (January 2024)

Species	Complete	Single	Duplicated	Fragmented	Missing
*Apis mellifera*	97.7%	97.6%	0.1%	0.3%	2.0%
*Tetragonula carbonaria*	97.5%	97.2%	0.3%	0.3%	2.2%
*Melipona beecheii*	97.4%	97.2%	0.2%	0.5%	2.1%
*Bombus terrestris*	96.5%	96.3%	0.2%	1.2%	2.3%
*Melipona quadrifasciata*	96.5%	96.3%	0.2%	1.2%	2.3%
*Frieseomelitta varia*	96.4%	96.2%	0.2%	1.2%	2.4%
***Melipona bicolor***	**96.3%**	**95.9%**	**0.4%**	**0.5%**	**3.2%**
*Heterotrigona itama*	93.3%	93.1%	0.2%	3.2%	3.5%
*Tetragonula mellipes*	88.8%	88.5%	0.3%	5.5%	5.7%
*Lepidotrigona ventralis hoosana*	88.5%	88.1%	0.4%	5.7%	5.8%
*Tetragonula davenporti*	87.9%	87.6%	0.3%	6.3%	5.8%
*Tetragonula clypearis*	85.7%	85.4%	0.3%	7.5%	6.8%
*Tetragonula hockingsi*	85.4%	85.1%	0.3%	7.3%	7.3%

### ***M. bicolor*** genome annotation

**Fig. 3:**
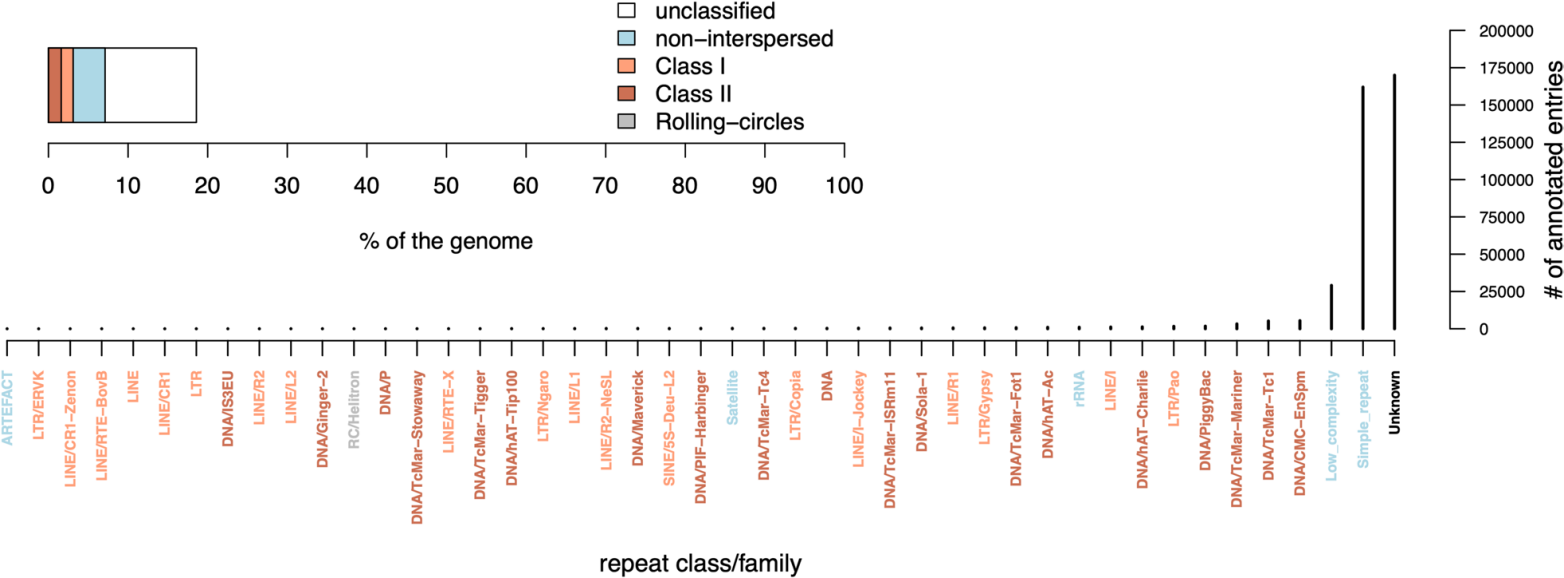
Repetitive elements found in the genome of *M. bicolor*. Repetitive regions accounted for about 18% of the genome assembly (upper plot). The lower plot shows the frequencies of all identified repeats with more frequent categories closer to the y-axis

Repetitive regions accounted for 18% of the genome. Most repeats were unclassified (11.5%) or non-interspersed (4%), while transposable elements of Class 1 and Class 2 corresponded to 1.5% and 1.6%, respectively. The complete classification of repetitive elements observed in the genome follows in Fig. 3. The repetitive elements final library and report are available at (https://github.com/nat2bee/repetitive_elements_pipeline/tree/master/database). Transcriptomic data used for gene prediction is deposited at NCBI under the SRA accession SRX5527788 and it consisted of 327.5 million paired reads. After repeating masking, 20,428 gene models were identified in the genome resulting in the annotation of 21,371 protein-coding genes and 150 tRNAs. Functional annotations were obtained for 15,010 of these protein-coding genes using the Funnannotate pipeline, while 11,905 M*. bicolor* genes were blasted against bee genes in the UniRef90 database.

### Comparative genomics

**Fig. 4:**
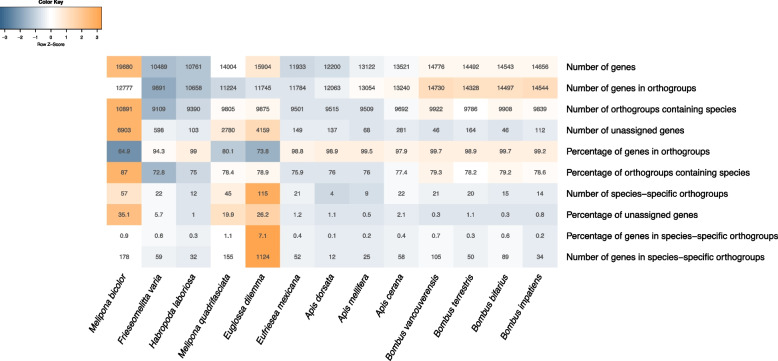
Summary results from OrthoFinder orthology analyses. Darker orange cells indicate larger values while darker shades of blue show smaller values compared to the others shown in the table

Among the twelve corbiculate bees (Table S1) and the outgroup species *H. laboriosa,* we found 12,514 orthogroups clustering 164,535 genes (91.4% of the total). The largest 4,440 orthogroups contained 14 or more genes, and 6,760 orthogroups included representative genes from all species, with 2,826 of these consisting of orthogroups of single-copy genes (all genes contained in orthogroups follow in Supplementary File 2). Although *M. bicolor* had the largest number of analyzed proteins, only 64.9% (or 12,777) of them were assigned to orthogroups (Fig. 4). Still, 87% of the orthogroups included *M. bicolor* genes and the proportion of species-specific genes for this species was not as high as that of other stingless bees (0.9%). Nineteen orthogroups were exclusive to eusocial corbiculate species and occurred in *M. bicolor* (Table S2), but not necessarily in other stingless bee genomes (15 also occurred in *M. quadrifasciata*, and 8 in *F. varia*). These orthogroups were associated with biological functions related to *nucleosome assembly*, *phospholipid metabolic process*, *lipid catabolic process*, *defense response to bacterium*, and *arachidonic acid secretion*.

**Fig. 5:**
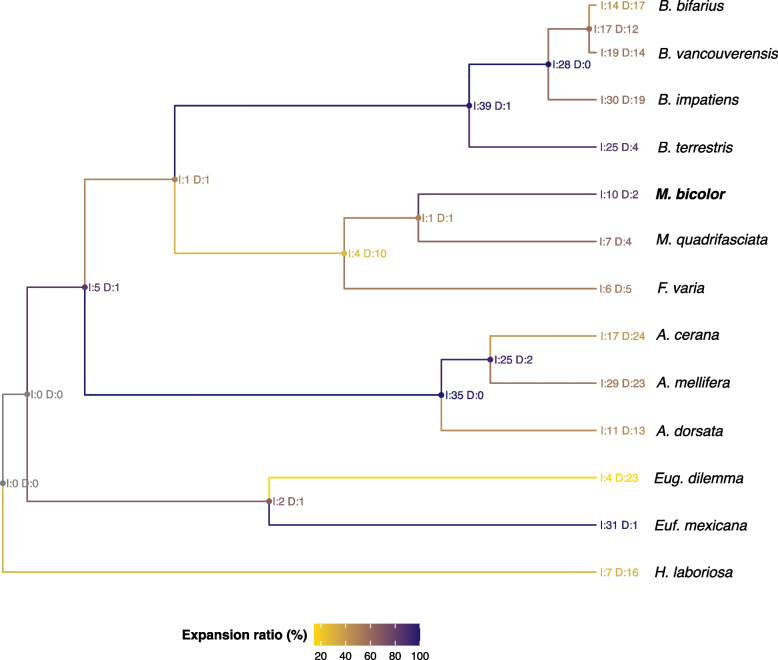
Ultrametric tree of the studied bee species indicating the significant gene family changes in each node. I = gene family expansion events (increase), D = gene family contraction events (decrease). Colors (from yellow to purple) indicate the proportion of changes that were due to gene family expansion, i.e., the expansion ratio is given by E = (D × 100) / (I + D). Nodes experiencing more gene family contractions are yellow while a larger proportion of expansion events is illustrated in purple. *M. bicolor* is highlighted in bold

Based on the 6,760 orthogroups including all species the estimated ultrametric species tree rooted at *H. laboriosa* had an average distance from root to leaves of 0.141434 (Supplementary File 1). Based on this tree and 12,548 orthologous genes (after excluding genes from large gene families) changes in gene family numbers (expansion or contraction) were identified. We found 148 gene families under significant changes across the studied species (p-value < 0.05, Supplementary File 3). Notably, in the nodes leading to the bumblebees and the honeybees, the great majority of changes led to increases in gene family sizes (Fig. 5). In the node leading to all eusocial corbiculates, five gene families associated with *carbohydrate metabolic process*, *lipid metabolic process*, *fatty acid biosynthetic process*, and *signal transduction* were found to be in expansion, while only one unknown gene family was reported to have contracted, and it did not have an associated function (Table S3). These results corroborate the relevance of alterations in metabolism and immunity in the evolution of eusocial corbiculate bees. The branch leading to *M. bicolor* had 12 gene families significantly changing (10 increasing and 2 decreasing, Fig. 5), these changes involved genes associated with the *fatty acid biosynthetic process*, *signal transduction*, and *feeding behavior* (Table S3).

**Fig. 6:**
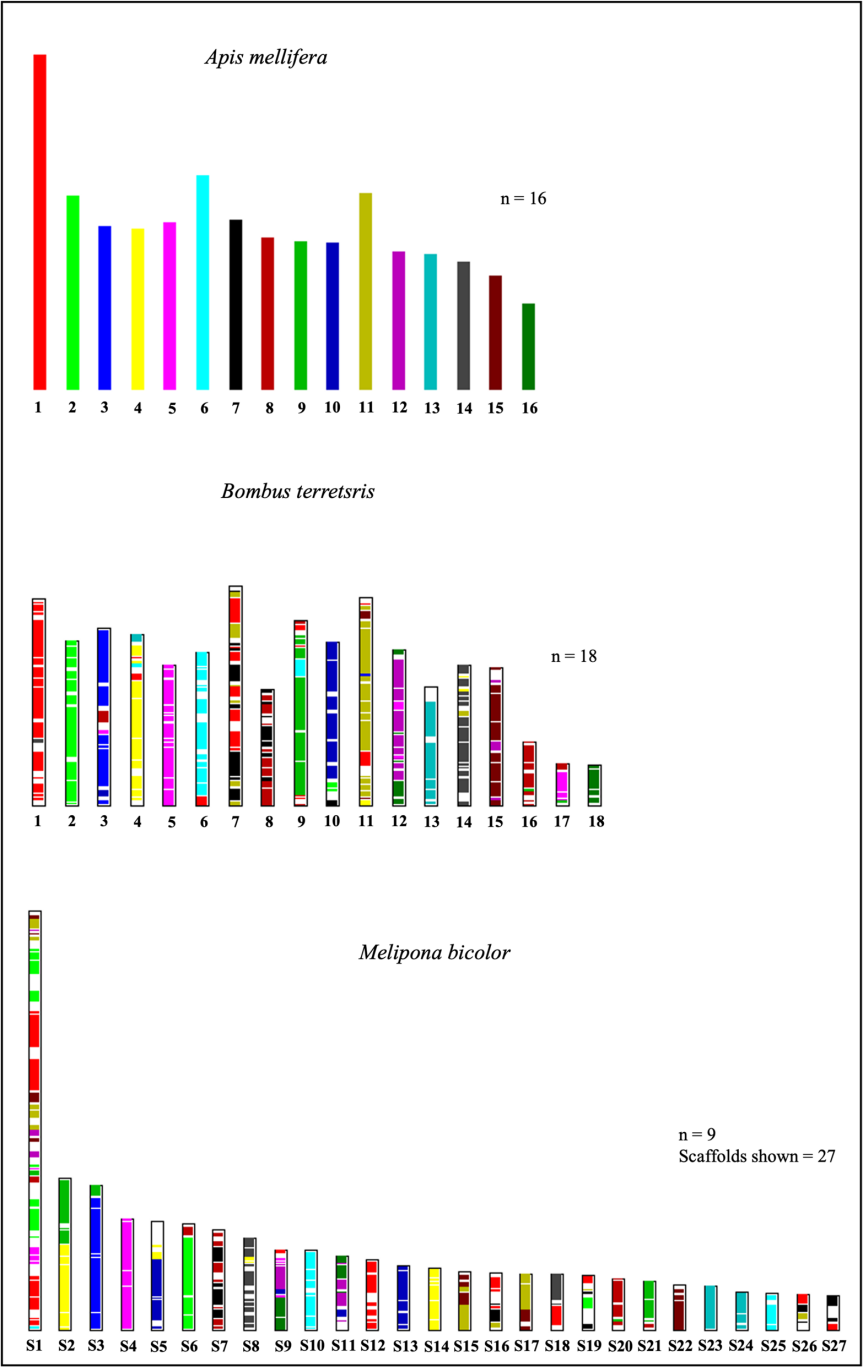
Genomic syntenies inferred among the genome assembly of *A. mellifera* (upper bars), *B. terrestris* (middle bars), and *M. bicolor* (lower bars). The comparisons shown use *A. mellifera* as the color reference, the same colors represent syntenic chromosome regions. For *M. bicolor* the largest 27 scaffolds are presented instead of chromosomes, representing 80% of the total genome assembly

Syntenic analyses among the genomes of *M. bicolor*, *B. terrestris,* and *A. mellifera* revealed multiple genome rearrangements across these eusocial corbiculate species (Fig. 6), with fewer events between *B. terrestris* and *M. bicolor* when compared to *A. mellifera* (Figures S4 and S5, respectively). This is explained by the closer phylogenetic relationship between *B. terrestris* and *M. bicolor* when compared to the honeybee as demonstrated previously through molecular phylogenies [27]. The long scaffold (S1) found in *M. bicolor* is likely correspondent to the larger chromosome 1 [12], as syntenic regions show it has several similarities with the honeybee and the bumblebee chromosome 1. Interestingly, this chromosome also includes syntenic regions in tandem with several chromosomes in the other two species (Fig. 6 and S4), suggesting it has gone through multiple rearrangements in Meliponini.

### Evolution of venom genes in stingless bees

We investigated the molecular evolution of 11 venom protein genes (*venom carboxylesterase-6-like*, *venom dipeptidyl peptidase 4*, *venom serine carboxypeptidase*, *venom peptide isomerase*, *c1q-like venom protein*, *cysteine-rich venom protein*, *venom protease-like*, *venom allergen* 5, *toxin 3FTx-Lei1*, *venom serine protease*, and *hyaluronidase*) by estimating synonymous and non-synonymous nucleotide changes and patterns of selection (d_N_, d_S,_ and ω values) focused on the Meliponini ancestral node. In the *branch* test, we found all venom genes tested were under negative or purifying selection in all the species studied, including all stingless bees (i.e., **ω** < 1 Table S4). Only two genes, the *venom dipeptidyl peptidase 4* and the *cysteine-rich venom protein*, significantly differed in Meliponini under the *b_free* evolutionary model. The *venom dipeptidyl peptidase 4*, a gene related to toxin maturation [28], had a larger **ω** value than the background data [**ω** (f) = 0,302 e **ω** (b) = 0,172] indicating a less intense negative selection in this gene. Conversely, the *cysteine-rich venom protein* showed the opposite pattern [**ω** (f) = 0,018 e **ω** (b) = 0,179] suggesting it could be under stronger positive selection within the Meliponini. The remaining nine venom genes tested were not significantly divergent in the Meliponini. Under the *branch-site* test, only the gene *venom dipeptidyl peptidase 4* was identified with a diverging evolutionary pattern in Meliponini, which suggests codon purifying selection is less intense in this gene among stingless bees, in agreement with the *branch* test.

## Discussion

The stingless bees comprise the most diverse group of highly eusocial bees, they have significant economic and ecological relevance in natural regions and show a range of unique ecological and behavioral adaptations. Still, genomic studies in stingless bees are scarce and we are far from understanding and characterizing the molecular diversity of these bees. Herein, we present a high-quality assembly for the genome of *Melipona bicolor*, this was the first stingless bee genome assembly released based on long-read sequencing data, allowing us to recover chromosome length scaffolds. *M. bicolor* genome was followed at the NCBI database a few months later by two stingless bee assemblies that also relied on long-read data providing high-quality genome assemblies for the bees *Melipona beecheii* and *Tetragonula carbonaria*. Besides representing a valuable reference for future molecular studies, these assemblies contribute to improving our understanding of the genomic diversity of eusocial corbiculate bees, especially as our orthologous analyses suggest stingless bees' genes might be underestimated in databases.

Syntenic analyses among eusocial corbiculate species and *M. bicolor* have shown interesting evolutionary patterns, such as several chromosome rearrangements among these lineages (Fig. 6). Based on the syntenic blocks, we found the longest scaffold in the genome of *M. bicolor* (lengthening 36 Mb) corresponds to chromosome 1 of the other bee genomes compared. Cytogenetic studies in *Melipona* have already shown that chromosome 1 is indeed the longest in most species of this genus – including *M. bicolor* [12, 29]. Here we found that scaffold 1 in *M. bicolor* encompasses regions syntenic to several chromosomes in *B. terrestris* and *A. mellifera*, especially to chromosomes one, two, and eleven (Figures S4 and S5). The coverage and mapping quality of long reads to this scaffold is rather good (Figure S1) strongly suggesting these results are not due to an assembly error. Instead, we argue this finding supports the hypothesis that multiple rearrangements involving several chromosomes occurred during the evolution of the *Melipona* genus resulting in its reduced haploid chromosome number [30]. It is unknown though how conserved this chromosome structure is across the genus, once size variation has been observed and heterochromatin distribution is remarkably variable across *Melipona* species [12, 29].

Compared to the other bees studied here, our gene annotation included more genes and over 35% of these genes were not assigned to Orthogroups. This may indicate that either we have identified many false positive genes in our genome assemblage, or the gene set of other bees is underrepresented for Meliponini genes. Notably, 15,010 genes in *M. bicolor* had a functional annotation, but only 12,777 genes were assigned to orthogroups. This supports the hypothesis that at least some of the genes in *M. bicolor* are real genes simply not reported in the other bee species, as they have received functional annotations. Moreover, through the BlobToolKit analyses, we found that some contigs significantly blasted to non-corbiculate bees from the Megachilidae and the Andrenidae families (Figure S3). This suggests that including these more distant lineages could have increased orthologous identification. Similarly, the genome of *M. quadrifasciata* also had one of the smaller proportions of genes included in orthogroups (80.1%), and like in *M. bicolor*, these non-orthologous proteins could represent prediction errors. The BUSCO results suggest that the genome of *M. quadrifasciata* is highly complete, still, this assembly is largely fragmented in 38,604 contigs with an N50 of 12,520 bp. The fragmentation of this genome can hinder gene annotation and consequently, orthologous identification between the two *Melipona* explaining, at least partially, the reduced number of proteins from these bees included in orthogroups. To delve further into this subject, we need a better genomic representation of the clade. We expect that as new high-quality bee genomes are sequenced the number of genes and orthologs found across species will also increase. Consequently, we argue that improving the completeness and reducing fragmentation of reference genomes, especially in stingless bee genomes, will affect the identification of adaptative orthologous. As the number of genes predicted in our annotation is considerably higher than what has been reported for other bee genomes (Fig. 4), in the absence of further evidence to support our predictions, we recommend that *M. bicolor* genes with no annotation and/or not assigned to orthogroups should be regarded carefully, as these genes have less support and could be originated from prediction errors. Accordingly, we have excluded these genes from our comparative analyses by focusing only on genes included within orthogroups, i.e. only 64.9% (12,777) of all *M. bicolor* genes were included in comparative analyses, and all considered, our orthologous comparisons should be regarded as preliminary at this stage.

In the eusocial corbiculate genomes (including Apini, Bombini, and *M. bicolor*), we found 19 exclusive ortholog families that did not occur in Euglossini and *H. laboriosa*. These were related to *nucleosome assembly*, *phospholipid metabolic process*, *lipid catabolic process*, *defense response to bacterium*, and *arachidonic acid secretion*. Additionally, through the gene family change analyses, we found that five gene families were significantly expanding in all eusocial corbiculate. These expanding gene families were related to carbohydrate and lipidic metabolism, and to signaling transduction, which included a gene family of odorant receptor genes. In agreement with these findings, adaptations in the metabolic and energetic pathways have been previously reported to be involved with eusocial adaptations in multiple studies [31–35]. We also found a few lineage-specific genes that should be further investigated for validation and further exploration of species adaptations, such as polygyny in *M. bicolor*. This includes 178 genes in species-specific orthogroups found only in *M. bicolor* and 12 gene families that significantly varied in size and included genes involved with the *fatty acid biosynthetic process*, *signal transduction*, and *feeding behavior.*

The maintenance of venom genes in stingless bee genomes is an intriguing evolutionary question. Although the melittin has been lost in this group, as observed by Koludarov et al. [28] and here, other 11 protein-related genes are still present in their genomes even though stingless bees (as the name suggests) do not have the morphological apparatus necessary for venom injection [9]. In addition, we found that most of these venom genes are still under purifying selection in stingless bees. Only two of the tested genes have significantly diverging molecular evolutionary patterns in stingless bees, and only one of them, the *venom dipeptidyl peptidase 4* is – as it would be expected – evolving through less intense purifying selection in stingless bees when compared to the other stinging species. Unexpectedly, the *cysteine-rich venom protein* showed an even stronger purifying selection in *Meliponini*. The *venom dipeptidyl peptidase 4* is related to toxin maturation [28]. In snakes, the *cysteine-rich venom protein* is involved with the inhibition of smooth muscle contraction and cyclic nucleotide-gated ion channels [36], but its function in bee venom is unknown. Koludarov et al. [28] suggested that most bee venoms may have been co-opted from other physiological functions and that in stingless bees, most of these genes are still present because they would be biologically relevant for other functions, even though they might have lost their venomous role. These findings are corroborated by our analyses, as we found that most venom genes are still evolving under purifying selection in stingless bees, supporting the hypothesis that they would be involved with alternative non-venom-related functions.

## Conclusions

Herein we provide the complete nuclear genome of the stingless bee *Melipona bicolor* that along with the complete mitochondrial genome previously published in [37] completes the genomic description of *M. bicolor*, the only true polygyne bee recognized so far. To illustrate the relevance of this dataset, we performed comparative analyses among the corbiculate bees unrevealing broad patterns of genomic evolution across this clade, including chromosomal rearrangements, gene family expansions, and lineage-specific genes. Finally, we found that the conserved repertoire of venom component genes remains under purifying selection in stingless bees despite their inability to sting. These findings contribute to our understanding of the molecular diversity and adaptation within stingless bees, and as the first high-quality genome available for this group, we believe this data will represent a useful source for future studies.

## Methods

### Data sequencing and genome assembly

We sampled *Melipona bicolor* from colonies kept at the bee lab at the University of São Paulo, São Paulo Campus – Brazil. For the genome sequencing, we used two haploid male pupae that were collected by opening reproductive cells within one monogynic colony. The males were recognized due to their characteristic head and eye shape (smaller heads and larger eyes than workers). Samples were immediately frozen in liquid nitrogen upon collection and kept at -20 °C until DNA extraction through a phenol/chloroform purification protocol (from step 8 as described in [38]). One male pupa was sequenced in the Illumina platform for paired reads of 100 bp using the TruSeq DNA PCR-Free Fit Library preparation kit. The second male was sequenced using the Pacbio Sequel SMRT Cell 1 M and the Sequencing Binding Kit 1.0 to generate long reads. Both sequencing strategies and library preparations were performed by Macrogen at their South Korea facility. For annotation purposes, we additionally sequenced the transcriptome of three females aged between 12–14 days. Upon their emergence, females were color-marked and reintroduced into the colony. After 12–14 days, we retrieved the marked females from the colony and immediately froze them in liquid nitrogen. During this stage of development, female workers are expected to primarily engage in tasks within the colony and are commonly referred to as nurses [39]. Total RNA from three female whole bodies was pooled for RNASeq at the Illumina HiSeq 2500 platform to generate 100 bp paired reads. The RNASeq was performed at Lactad—Unicamp. Then, the de novo transcriptome assembly was performed using Trinity v2.8.4 [40] to be used in the genome annotation. The genome assembly was initially performed in two ways, first using Falcon v1.3.0 [41] with only the long-read data, and secondly using MaSuRCA v3.3.5 [42] based on a combination of short and long reads. Parameters used in the Falcon assembly were: *genome_size* = *0; seed_coverage* = *20; length_cutoff* = *1000; ovlp_daligner_option* = *–e.93 -l2500 -k24 -h1024 -w6 -s100*. MaSuRCA run was set up for a k-mer size of 67 for the graph step and 25 × coverage for the longest reads. Both assemblies were corrected using arrow (Pacbio tools gcpp v1.9.0) with default parameters after realignment of the long reads with pbmm2 v1.2.0. After polishing, the two assemblies were combined using quickmerge wrapper v0.3 with MUMmer v3.23 [43] with the parameter *-l 721765*. We then removed the mitochondrial contig from the assembly by aligning *M. bicolor* mitogenome [37] to the assembly using Last v1047 [44] with the options *-uNEAR -R01*. For further scaffolding, we used the transcriptome assembly and the program SCUBAT v2 [45] with 40,000 bp as the maximum intron size, since 99% of all introns assembled had a maximum size of 38,984 bp. The resulting assembly was polished with arrow again, using only the long reads and default parameters, then with pilon v1.23 [46] using the parameter *-Xmx250G* and the short reads. We then re-aligned the long and short reads using pbmm2 and bwa v0.7.17 [47], respectively, to the polished assembly and analyzed the quality of the alignments using Qualimap v2.2.1 [48]. Based on this, we removed contigs with extreme coverage bias (i.e., with mean coverage ≤ 1) according to long reads alignment, and one contig that had a very high coverage and aligned to the mitogenome (GC content 40%). The removal of these contigs did not affect BUSCO quality scores. Lastly, an additional polishing step using short reads and long reads combined was performed using hypo v1.0.3 [49]. For quality estimation throughout the assembly processes, we have used QUAST v5.0.2 [50], BUSCO v5.1.2 [51], Qualimap, and BlobToolKit v4.1.5 [52] with default parameters for genomic analyses.

### Genome annotation

Repetitive elements were identified in the genome using the pipeline available at https://github.com/nat2bee/repetitive_elements_pipeline. Briefly, we used RepeatModeler v1.0.11 [53], TransposonPSI [54], and LTRharvest from GenomeTools v1.6.1 [55] to build custom repeat libraries. These libraries were merged into a single non-redundant library of repetitive elements for *M. bicolor* using USEARCH v11.0.667 [56] (< 80% identity). RepeatClassifier was used for the library annotation. Then, we concatenated our custom library with the Dfam v3.1 Hymenoptera library included in RepeatMasker v4.1.0 [57] and used the same program to annotate and soft mask the repeats found in the genome based on our custom library (available at https://github.com/nat2bee/repetitive_elements_pipeline). Repeats Summary statistics of the annotated repeats were obtained using a custom script *RepeatMasker_stats.py* (https://github.com/nat2bee/repetitive_elements_pipeline). Genome annotation of non-masked regions was performed using Funannotate v1.7.4 pipeline [58] which combined gene predictions based on *M. bicolor *de novo transcriptomic assembly (9,458 gene models) and the Insecta gene model database from BUSCO (all database versions are detailed in Supplementary File 1). Functional annotation of the genes was performed also under Funannotate in tandem with InterProScan5. *M. bicolor* genes were additionally blasted, using NCBI-blatsp [59], against bee proteins in the UniRef90 database (from August 2021). From these results, gene names were preferentially assigned and the gene ontology terms (GO) were retrieved. Finally, the annotation and the genome were trimmed according to NCBI’s quality filtering. Unless stated in the text, the parameters used were the program's suggested default.

### Comparative genomics

We compared the genome assembly of *M. bicolor* with that of other eleven corbiculate bees (*Apis cerana*, *A. dorsata*, *A. mellifera*, *Bombus bifarius*, *B. impatiens*, *B. terrestris*, *B. vancouverensis*, *Eufriesea mexicana*, *Euglossa dilemma*, *Frieseomelitta varia*, and *Melipona quadrifasciata*) and one outgroup species (*Habropoda laboriosa*) using OrthoFinder v2.5.4 [60] and CAFE v5.0.0 [61]. These genomes were chosen because they comprise lineages representing all corbiculate clades and were available in the NCBI database along with their attributed gene annotations (Table S1), except for *E. dilemma* whose genome annotation was retrieved from the Beebase (https://hymenoptera.elsiklab.missouri.edu/beebase/consortium_datasets) so we could include at least two species per clade. For the genomes in which the RefSeq annotation was not available (i.e., for *E. dilemma, F. varia*, *M. quadrifasciata*, and *M. bicolor*), we selected one isoform per gene by grouping similar proteins using CD-Hit V4.8.1 [62] with a 70% similarity threshold. Using OrthoFinder with default parameters we generated the species tree and re-rooted it at *H. laboriosa* to identify orthologs and species-specific genes across all branches. We then generated an ultrametric tree using the OrthoFinder *make_ultrametric.py* script and calibrated the tree root node at 105 million years [27]. We identified gene family changes across the tree nodes using CAFE. We initially removed the two largest gene families using the *lade_and_size_filter.py* script and then estimated the parameters to run CAFE. First by measuring the error parameter to account for possible genome assembly errors, then by testing multiple k values (from 1 to 10) to choose the one with the highest likelihood score, and finally, by running the estimates multiple times and getting the median lambda values. We finally run CAFE using the command *-eerror_model_017.txt -l 0.0017312* where the error model represents a text file containing the following information: *maxcnt: 105; cntdiff: -1 0 1; 0 0 0.982961 0.0170389; 1 0.0170389 0.965922 0.0170389*.

Since *M. bicolor* assembly resulted in large contigs and N50, we inferred the synteny between this genome and two high-quality genome assemblies representing the other eusocial corbiculate bee lineages – *i.e.*, the Apini (*A. mellifera*) and Bombini (*B. terrestris*) – both assemblies with associated chromosome mappings. For this analysis, we first blasted species protein sets with each other using blastp from NCBI-blast + v2.9.0 [59] with the parameters *-e 1e-10 -b 5 -v 5 -m 8*, and then used the MCScanX [63] with default parameters to identify and illustrate collinear blocks across species. Since the genome of *M. bicolor* is not assembled to the chromosome level, we inferred the synteny based on the 27 largest scaffolds, which corresponds to 80% of the genome.

### Evolution of venom genes in stingless bees

To study the evolution of venom-related genes in stingless bees, we selected the venom-associated genes based on [28] findings. These authors focused on 12 proteins prevalent in the bee venom. Among these, we excluded the *melittin* gene from our analysis because it is not present in the stingless bee genomes (including in *M. bicolor*). Orthogroups containing the remaining 11 venom protein genes (i.e., *venom carboxylesterase-6-like*, *venom dipeptidyl peptidase 4*, *venom serine carboxypeptidase*, *venom peptide isomerase*, *c1q-like venom protein*, *cysteine-rich venom protein*, *venom protease-like*, *venom allergen* 5, *toxin 3FTx-Lei1*, *venom serine protease*, and *hyaluronidase*) were filtered from OrthoFinder results obtained in the comparative analyses. To select only a single gene copy per orthogroup we aligned all sequences included in the orthogroup using Mafft v7.310 [64] – with the parameter *adjustdirectionaccurately* – and selected the best-aligning gene copy (*i.e.*, the copy whose alignment produced fewer gaps in comparison to the other bee species).

The selected amino acid sequences from all species were aligned with Mafft and posteriorly polished using Gblocks v0.91b [65]. Molecular evolution tests supported by species phylogeny were performed using CODEML within the PAML4 package [66] to estimate synonymous and non-synonymous nucleotide changes (d_N_, d_S,_ and ω values). ETE3 v3.1.2 [67] was used to automate these analyses. The Meliponini ancestral node was used as the foreground node to focus on the evolutionary pattern of venom genes in this branch. We tested six different evolutionary models for each gene, three using the *branch* (*M0*, *b_neut*, and *b_free*) and three using the *branch-site* (*M1*, *bsA1* e *bsA*) model. The *branch* model tests estimate the molecular evolution of the entire gene in the foreground node compared to the remaining (background) nodes, while the branch-site models search for codon positive selection in the foreground node [66]. Model results were interpreted according to likelihood ratio tests and associated *p*-values, which were corrected for multiple comparisons with a cut-off of *p* < 0.05.

### Supplementary Information

**Additional file 1: Table S1.** List of bee genome assemblies used for comparative analyses. **Table S2.** Orthogroups exclusive to eusocial corbiculate bees and containing M. bicolor. **Table S3.** Gene families under significant changes (expanding or contracting) in the nodes of all eusocial corbiculate bees and the node of M. bicolor. **Table S4.** Results of the selection tests of the genes associated with the bee venom using the Meliponini ancestral node as the foreground node. The branch model tests compare the entire gene evolution in the foreground (f) node to the remaining background (b) nodes, while the branch-site models search for codon selection in the foreground node. Significant values are highlighted in bold. **Figure S1.** Sequencing coverage and mapping quality of the alignment of short reads (upper plot) and long reads (lower plot) against the final genome assembly of M. bicolor. Below each coverage plot the mean GC content is shown and 400 windows were used to represent the genome regions. Each scaffold is represented by a segment that are separated by dotted lines. **Figure S2.** Sequencing coverage of short reads (x-axis) against long reads (y-axis) showing that coverage of the assembled scaffolds was mostly congruent between the two sequencing technologies as we removed outlier scaffolds (i.e., scaffolds with coverage <1x) during quality trimming steps. Circle size represents scaffold size. **Figure S3.** Blob plot of short reads base coverage against the GC proportion of scaffolds in the genome assembly of Melipona bicolor. Sequences are colored by matching genus sequences in databases. Circle size represents scaffold size. The histograms show the sequence length distribution in each axis. **Figure S4.** Genomic synteny inferred between the genome assembly of B. terrestris (upper bars), and M. bicolor (lower bars). The same colors represent syntenic chromosome regions in the B. terrestris genome. For M. bicolor the largest 27 scaffolds are presented instead of the chromosomes, these represent 80% of the total genome assembly. **Figure S5.** Genomic synteny inferred between the genome assembly of A. mellifera (upper bars), and M. bicolor (lower bars). The same colors represent syntenic chromosome regions in the A. mellifera genome. For M. bicolor the largest 27 scaffolds are presented instead of the chromosomes, these represent 80% of the total genome assembly.

**Additional file 2.** Genes containned in all orthogroups identified across species.

**Additional file 3.** Gene Families under changes in size across the studied nodes. Positive values indicate gains, negative losses.

## Data Availability

The datasets generated and/or analyzed during the current study are available in the NCBI repository, BioProject PRJNA632864, and accession code JAHYIQ01.
