# Supplementary material for: Insights from Melipona bicolor hybrid genome assembly: a stingless bee genome with chromosome-level scaffold
Source: BMC Genomics. 2024 Feb 13;25:171. doi: 10.1186/s12864-024-10075-x (PMC10863234; doi:10.1186/s12864-024-10075-x)
Supplement: Supplementary file 1 — Additional file 1: Table S1. List of bee genome assemblies used for comparative analyses. Table S2. Orthogroups exclusive to eusocial corbiculate bees and containing M. bicolor. Table S3. Gene families under significant changes (expanding or contracting) in the nodes of all eusocial corbiculate bees and the node of M. bicolor. Table S4. Results of the selection tests of the genes associated with the bee venom using the Meliponini ancestral node as the foreground node. The branch model tests compare the entire gene evolution in the foreground (f) node to the remaining background (b) nodes, while the branch-site models search for codon selection in the foreground node. Significant values are highlighted in bold. Figure S1. Sequencing coverage and mapping quality of the alignment of short reads (upper plot) and long reads (lower plot) against the final genome assembly of M. bicolor. Below each coverage plot the mean GC content is shown and 400 windows were used to represent the genome regions. Each scaffold is represented by a segment that are separated by dotted lines. Figure S2. Sequencing coverage of short reads (x-axis) against long reads (y-axis) showing that coverage of the assembled scaffolds was mostly congruent between the two sequencing technologies as we removed outlier scaffolds (i.e., scaffolds with coverage <1x) during quality trimming steps. Circle size represents scaffold size. Figure S3. Blob plot of short reads base coverage against the GC proportion of scaffolds in the genome assembly of Melipona bicolor. Sequences are colored by matching genus sequences in databases. Circle size represents scaffold size. The histograms show the sequence length distribution in each axis. Figure S4. Genomic synteny inferred between the genome assembly of B. terrestris (upper bars), and M. bicolor (lower bars). The same colors represent syntenic chromosome regions in the B. terrestris genome. For M. bicolor the largest 27 scaffolds are presented instead of the chromoso [file 12864_2024_10075_MOESM1_ESM.pdf]

Supplementary information

1- Databases used for gene evidence in the Funannotate pipeline.

- MEROPS Database: version=12.0 date=2017-10-04 records=5,009
- UniProtKB Database: version=2021_03 date=2021-06-02 records=565,254
- dbCAN Database: version=9.0 date=2020-08-04 records=607
- Pfam Database: version=34.0 date=2021-03 records=19,179
- Repeat Database: version=1.0 date=2021-07-28 records=11,950
- GO ontology version=2021-07-02 date=2021-07-02 records=47,228
- InterProScan XML: version=86.0 date=2021-06-03 records=38,913
- BUSCO outgroups: version=1.0 date=2021-07-28 records=8
- Gene2Product: version=1.70 date=2021-06-15 records=34,039
- Downloading busco models: insecta

2- Ultrametric tree calculated for the studied species in Newick format.

(((Eufriesea_mexicana:57.8594,Euglossa_dilemma:57.8594):42.8287,(((Bombus_terrestris:22.4391,(Bombus_impatiens:8.42918,(Bombus_vancouverensis:1.21064,Bombus_bifarius:1.21064):7.21854):14.0099):52.1207,(Frieseomelitta_varia:44.5763,(Melipona_quadrifasciata:31.4319,Melipona_bicolor:31.4319):13.1444):29.9835):15.9248,((Apis_mellifera:18.7074,Apis_cerana:18.7074):8.68206,Apis_dorsata:27.3895):63.0952):10.2034):4.31195,Habropoda_laboriosa:105);

Supplementary Tables

Table SI. List of bee genome assemblies used for comparative analyses.

Species	NCBI acession	Scaffolds	Contigs	Length (Mb)	CDS	Scaffolds N50	Scaffolds L50	N in 100 kbp (%)	Reference
Apis cerana	GCF_001442555.1	2,430	10,706	228.3	20,933	1.4 Mb	42	2.31	Park et al. (2015) *BMC genomics*. DOI: 10.1186/1471-2164-16-1
Apis dorsata	GCF_000469605.1	4,040	45,204	230.3	20,012	732.1 kb	87	4.95	Fouks et al. (2021) *Genome Res.* DOI: 10.1101/gr.272310.120
Apis mellifera	GCF_003254395.2	176	227	225.2	23,471	13.6 Mb	7	0	Wallberg et al. (2019) *BMC Genomics*. DOI: 10.1186/s12864-019-5642-0
Bombus bifarius	GCF_011952205.1	1,249	1,251	266.8	23,896	2.2 Mb	30	0	Heraghty et al. (2020) *G3*. DOI: 10.1534/g3.120.401437
Bombus impatiens	GCF_000188095.3	5,460	16,060	246.9	24471	1.4 Mb	54	0.79	Sad et al. (2015) *Genome Biol.* DOI: 10.1186/s13059-015-0623-3
Bombus terrestris	GCF_000214255.1	5,678	10,672	248.7	22,091	3.5 Mb	18	0.24	Sad et al. (2015) *Genome Biol.* DOI: 10.1186/s13059-015-0623-3
Bombus vancouverensis	GCF_011952275.1	1,162	1,164	282.1	24,285	3.1 Mb	24	0	Heraghty et al. (2020) *G3*. DOI: 10.1534/g3.120.401437
Eufriesea mexicana	GCF_001483705.1	187,611	212,650	596.3	15,659	351.9 kb	377	0	Kapheim et al. (2015) *Science*. DOI: 10.1126/science.aaa4788
Euglossa dilemma	GCA_002201625.1	22,698	110,426	588.2	15,904	143.6 kb	419	5.3	Brand et al. (2017) *G3.* DOI: 10.1534/g3.117.043687
Frieseomelitta varia	GCF_011392965.1	2,173	6,729	275.4	23,628	467 kb	176	1.12	Freitas et al. (2020) *BMC Genomics*. DOI: 10.1186/s12864-020-06784-8
Habropoda laboriosa	GCF_001263275.1	27,566	50,205	297	12,256	1.8 Mb	47	0	Kapheim et al. (2015) *Science*. DOI: 10.1126/science.aaa4788
Melipona bicolor	GCA_030673865.1	241	244	259.9	20,278	6.2 Mb	12	0	This study
Melipona quadrifasciata	GCA_001276565.1	2,866	38,604	256.3	14,257	1.9 Mb	42	0	Kapheim et al. (2015) *Science*. DOI: 10.1126/science.aaa4788
Tetragonula carbonaria	GCA_010645115.1	NA	90201	299.6	NA	13.6 kb	5949	0	NA
Tetragonula clypearis	GCA_010645135.1	NA	77144	294	NA	14.9 kb	5525	0	NA
Tetragonula davenporti	GCA_010645165.1	NA	47286	284.6	NA	18.5 kb	4237	0	NA
Tetragonula hockingsi	GCA_010645185.1	NA	112753	307.1	NA	10.5 kb	7605	0	NA
Tetragonula mellipes	GCA_011634685.1	NA	133257	337.6	NA	16.4 kb	5285	0	NA

Table SII. Orthogroups exclusive to eusocial corbiculate bees and containing *M. bicolor*.

HOG	*A. cerana*	*A. dorsata*	*A. mellifera*	*B. bifarius*	*B. impatiens*	*B. terrestris*	*B. vancouverensis*	*F. varia*	*M. bicolor*	*M. quadrifasciata*	Annotation in *M. bicolor*	GO in *M. bicolor*
N0.HOG0002289	XP_016914686.1, XP_016914687.1, XP_016913788.1	XP_031366627.1	XP_001120293.2, NP_001011614.1	XP_033319182.1, XP_033319184.1	XP_012248547.1, XP_012248546.1, XP_033180239.1	XP_012170987.1, XP_012170988.1	XP_033185943.1	KAF3421744.1, KAF3421743.1	K0M31_012811-T1	KOX69497.1	Phosphatidylcholine 2-acylhydrolase (EC 3.1.1.4)	GO:0006644, GO:0050482, GO:0016042, GO:0005576, GO:0102567, GO:0102568, GO:0004623, GO:0046872
N0.HOG0003494	XP_028522782.1, XP_028520410.1, XP_028520409.1, XP_016905282.1	XP_031370965.1	XP_026300628.1	XP_033319259.1	XP_024221752.1	XP_012171089.2	XP_033199797.1, XP_033182849.1		K0M31_009555-T1, K0M31_009554-T1	KOX67239.1, KOX67232.1	Uncharacterized protein	
N0.HOG0005010	XP_016919808.1	XP_031365150.1	XP_006568169.1, XP_624119.2	XP_033315548.1	XP_024220196.1	XP_012168725.1, XP_003398715.1	XP_033198891.1	KAF3430281.1, KAF3426545.1, KAF3429429.1	K0M31_002407-T1	KOX77185.1	Putative RNA-binding protein EEED8.10 (Fragment)	GO:0003723
N0.HOG0008548	XP_016904248.1	XP_031368500.1	XP_016766172.1	XP_033297622.1	XP_003487765.1	XP_012165659.1	XP_033183336.1	KAF3422505.1, KAF3422504.1	K0M31_006915-T1, K0M31_006914-T1	KOX73507.1	Uncharacterized protein	GO:0016021
N0.HOG0008949	XP_016916307.1	XP_006611907.1	NP_001011613.1	XP_033310662.1	XP_003491768.1, XP_033178254.1	XP_012175465.1	XP_033196951.1, XP_033196952.1	KAF3424888.1	K0M31_020235-T1	KOX78578.1	Apidaecins type 22	GO:0005576, GO:0042742
N0.HOG0009274	XP_028522658.1	XP_031367232.1, XP_031367231.1	XP_026295388.1, XP_026295434.1	XP_033306901.1	XP_033175218.1	XP_020724381.1, XP_020724377.1	XP_033204903.1		K0M31_011481-T1	KOX71343.1	MYND-type domain-containing protein	
N0.HOG0009323	XP_028525174.1	XP_031368067.1	XP_026300959.1, XP_026300958.1, XP_026300957.1, XP_006560249.3	XP_033320693.1	XP_033177771.1	XP_020722146.1	XP_033197784.1		K0M31_019774-T1	KOX71531.1	uncharacterized protein LOC110119859	
N0.HOG0009429	XP_016907290.2	XP_006609518.1	XP_026298210.1	XP_033303772.1	XP_012237456.1	XP_012169489.1	XP_033190083.1	KAF3420998.1	K0M31_002469-T1, K0M31_002470-T1	KOX77138.1	LIM/homeobox protein Awh	GO:0005634, GO:0046872, GO:0003677

N0.HOG0009691	XP_016904427.1	XP_031365192.1	XP_026301822.1	XP_033302516.1	XP_033180106.1	XP_020719386.1	XP_033183844.1		K0M31_005632-T1, K0M31_005631-T1	KOX77512.1	Uncharacterized protein,Cytoglobin-2	GO:0005344, GO:0019825, GO:0020037, GO:0005833, GO:0005576, GO:0046872
N0.HOG0009694	XP_016905072.1	XP_006610175.1	XP_006561909.1	XP_033298308.1	XP_012237621.1	XP_003397817.1	XP_033202021.1	KAF3423249.1	K0M31_013427-T1	KOX75389.1	Uncharacterized protein	
N0.HOG0009730	XP_016911234.1	XP_006609717.1	XP_026295425.1	XP_033301161.1	XP_024223348.1	XP_012176274.1	XP_033184964.1	KAF3425246.1	K0M31_011809-T1	KOX68466.1	Cytochrome c oxidase subunit 6A1, mitochondrial	GO:0004129, GO:0005751
N0.HOG0009760	XP_016922059.1, XP_028519992.1	XP_006610171.1	XP_026298543.1	XP_033298195.1	XP_012239961.1	XP_012167339.1	XP_033201885.1		K0M31_008604-T1	KOX74284.1	keratin-associated protein 10-8-like	
N0.HOG0009765	XP_028520355.1	XP_031369824.1	XP_006561149.2, XP_016766411.2	XP_033310493.1	XP_012248793.1	XP_012169860.2	XP_033198641.1		K0M31_000254-T1, K0M31_000255-T1		PDZ domain-containing protein 2	
N0.HOG0009897	XP_016913059.1, XP_016913058.1	XP_006613452.1	XP_026300237.1	XP_033308953.1	XP_024227403.1	XP_020721432.1	XP_033190734.1		K0M31_013097-T1		uncharacterized protein LOC100652324	
N0.HOG0009898	XP_016913182.1	XP_031368418.1	XP_006560911.2	XP_033309516.1	XP_012243091.1	XP_003397027.1	XP_033195976.1		K0M31_018409-T1	KOX70140.1	Uncharacterized protein	
N0.HOG0009911	XP_016917430.1	XP_006614906.1	XP_026295147.1	XP_033300664.1	XP_024223318.1	XP_020724099.1	XP_033184326.1		K0M31_013335-T1	KOX79962.1	uncharacterized protein LOC112212842	GO:0006334, GO:0003677, GO:0000786
N0.HOG0010049	XP_016909912.1	XP_031371339.1	XP_003250947.2	XP_033316157.1	XP_024225515.1	XP_020722860.1	XP_033198982.1		K0M31_008297-T1		Uncharacterized protein	
N0.HOG0010070	XP_016919457.1	XP_031366306.1	XP_016770809.1	XP_033315019.1	XP_012241182.1	XP_003398842.1	XP_033206859.1		K0M31_000208-T1		P17/29C-like protein DDB_G0287399	
N0.HOG0010098	XP_028525478.1	XP_031364702.1	XP_016769345.2	XP_033298352.1	XP_024221480.1	XP_020720473.1	XP_033202325.1		K0M31_008781-T1		uncharacterized protein LOC110119589	

Table SIII. Gene families under significant changes (expanding or contracting) in the nodes of all eusocial corbiculate bees and the node of *M. bicolor*.

Gene family ID	Genes annotation in *M. bicolor*	Gos in *M. bicolor*	Number of changes
**Family changes in the node of all eussocial corbiculate**			
N0.HOG0000029	Fatty acyl-CoA reductase (EC 1.2.1.84)	GO:0102965,GO:0016021,GO:0080019, GO:0006629	+1
N0.HOG0000059	null		-1
N0.HOG0000082	Putative odorant receptor 13a,Odorant receptor,Putative odorant receptor 92a	GO:0005549,GO:0016021,GO:0004984, GO:0007165,GO:0005886	+1
N0.HOG0000120	Lysophospholipase (EC 3.1.1.3) (EC 3.1.1.4) (EC 3.1.1.5) (Phospholipase A2) (Phospholipase B/lipase) (Phospholipase B1, membrane-associated, membrane-associated) (Triacylglycerol lipase),Phospholipase B1	GO:0004806,GO:0006629,GO:0016324, GO:0016021,GO:0102568,GO:0102567, GO:0004622,GO:0004623,GO:0004620, GO:0102545	+1
N0.HOG0000170	Maltase 2,Alpha-glucosidase	GO:0003824,GO:0016021,GO:0005975	+1
N0.HOG0000235	Elongation of very long chain fatty acids protein (EC 2.3.1.199) (Very-long-chain 3-oxoacyl-CoA synthase)	GO:0006633,GO:0016021,GO:0102337, GO:0102336,GO:0102338,GO:0102756	+1
**Family changes in the node of *M. bicolor***			
N0.HOG0000030	Zinc finger imprinted 3,Uncharacterized protein,Zinc finger protein,Zinc finger protein CKR1,longitudinals lacking protein isoform X37, isoforms A/B/D/L,Longitudinals lacking protein		+4
N0.HOG0000056	Uncharacterized protein	GO:0005549,GO:0016021,GO:0005886, GO:0004984	-2
N0.HOG0000108	Uncharacterized protein		+1
N0.HOG0000135	Uncharacterized protein	GO:0016021	+1
N0.HOG0000222	Acyltransferase (EC 2.3.1.-)	GO:0016021,GO:0016747,GO:0005789	+2
N0.HOG0000225	Putative odorant receptor 13a,Odorant receptor	GO:0005549,GO:0016021,GO:0004984, GO:0007165,GO:0005886	+4
N0.HOG0000235	Elongation of very long chain fatty acids protein (EC 2.3.1.199) (Very-long-chain 3-oxoacyl-CoA synthase)	GO:0006633,GO:0016021,GO:0102337, GO:0102336,GO:0102338,GO:0102756	-2
N0.HOG0000342	organic cation transporter protein-like	GO:0016021,GO:0022857	+1
N0.HOG0000404	LOW QUALITY PROTEIN: uncharacterized protein LOC100746456,LOW QUALITY PROTEIN: uncharacterized protein LOC117206071,Fibrillin-2	GO:0005634,GO:0005576,GO:0005509, GO:0005520,GO:0016021,GO:0008270	+2
N0.HOG0000561	Ig-like domain-containing protein (Fragment)		+1
N0.HOG0000685	null		+1
N0.HOG0000868	Orexin receptor type 2,allatotropin receptor isoform X1 (allatotropin receptor precursor)	GO:0007631,GO:0016021,GO:0005886, GO:0004983,GO:0016499	+1

Table SIV. Results of the selection tests of the genes associated with the bee venom using the Meliponini ancestral node as the foreground node. The *branch* model tests compare the entire gene evolution in the foreground (f) node to the remaining background (b) nodes, while the branch-site models search for codon selection in the foreground node. Significant values are highlighted in bold.

		branch					***branch-site***						
**Orthogroup**	**Gene**	**mean ω (*M0*)**	**ω (f)**	**ω (b)**	**p-value *M0***	**p-value *b-neut***	**mean ω (*M1*)**	**condon positions under selection**	**number of codons under selection**	**p-value *M1***	**p-value *bsA1***	**p-value *bsA-M1***	**p-value *bsA1-bsA***
N0.HOG0001460	*Venom carboxylesterase-6-like*	0,199	0,179	0,201	0,742	**0,000**	0,253	NA	0	1,000	1,000	0,974	0,822
N0.HOG0004097	*Venom dipeptidyl peptidase 4*	0,181	0,302	0,172	**0,016**	**0,000**	0,283	**62, 85, 316**	**3**	**0,000**	0,638	**0,000**	0,070
N0.HOG0004530	*Venom serine carboxypeptidase*	0,155	0,169	0,154	0,742	**0,000**	0,245	NA	0	1,000	1,000	0,980	1,000
N0.HOG0006230	*Venom peptide isomerase*	0,136	0,075	0,146	0,151	**0,000**	0,195	NA	0	1,000	1,000	1,000	1,000
N0.HOG0005126	*C1q-like venom protein*	0,221	0,090	0,258	0,151	**0,000**	0,294	NA	0	1,000	0,638	0,356	0,151
N0.HOG0000658	*Cysteine-rich venom protein*	0,156	0,018	0,179	**0,000**	**0,000**	0,205	NA	0	1,000	1,000	1,000	1,000
N0.HOG0004893	*Venom protease-like*	0,238	0,268	0,235	0,742	**0,000**	0,293	NA	0	1,000	0,647	0,471	0,245
N0.HOG0002840	*Venom allergen 5*	0,199	0,279	0,194	0,742	**0,046**	0,294	NA	0	1,000	1,000	1,000	1,000
N0.HOG0006648	*Toxin 3FTx-Lei1*	0,200	0,120	0,218	0,742	**0,030**	0,224	NA	0	1,000	1,000	1,000	1,000
N0.HOG0005748	*Venom serine protease*	0,296	0,333	0,292	0,742	**0,000**	0,403	NA	0	0,225	0,638	0,041	0,174
N0.HOG0004751	*Hyaluronidase isoform x1*	0,151	0,141	0,153	0,742	**0,000**	0,205	NA	0	1,000	0,647	0,576	0,294

Supplementary Figures

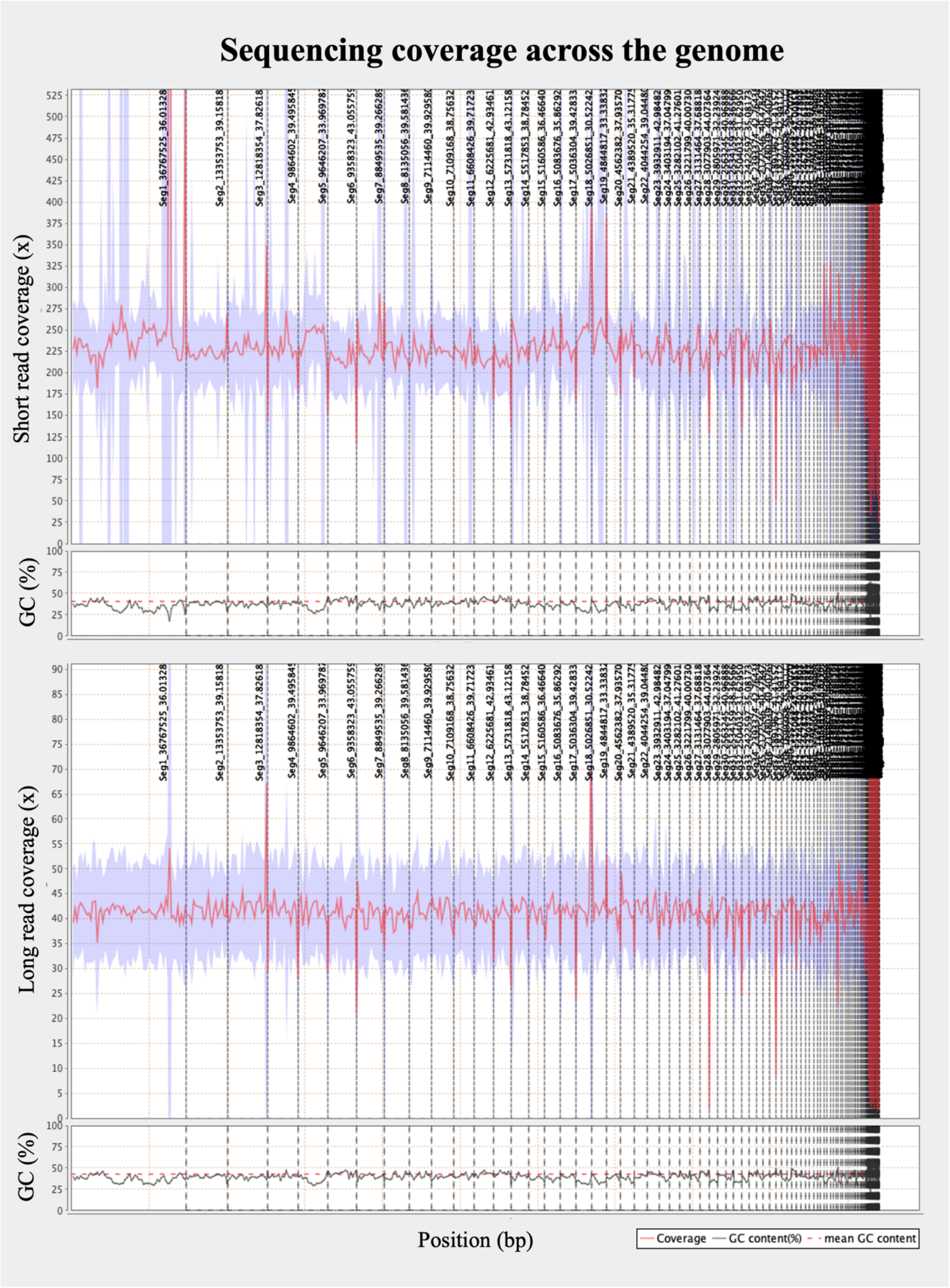

Figure S1 Sequencing coverage and mapping quality of the alignment of short reads (upper plot) and long reads (lower plot) against the final genome assembly of *M. bicolor*. Below each coverage plot the mean GC contest is shown and 400 windows were used to represent the genome regions. Each scaffold is represented by a segment that are separated by dotted lines.

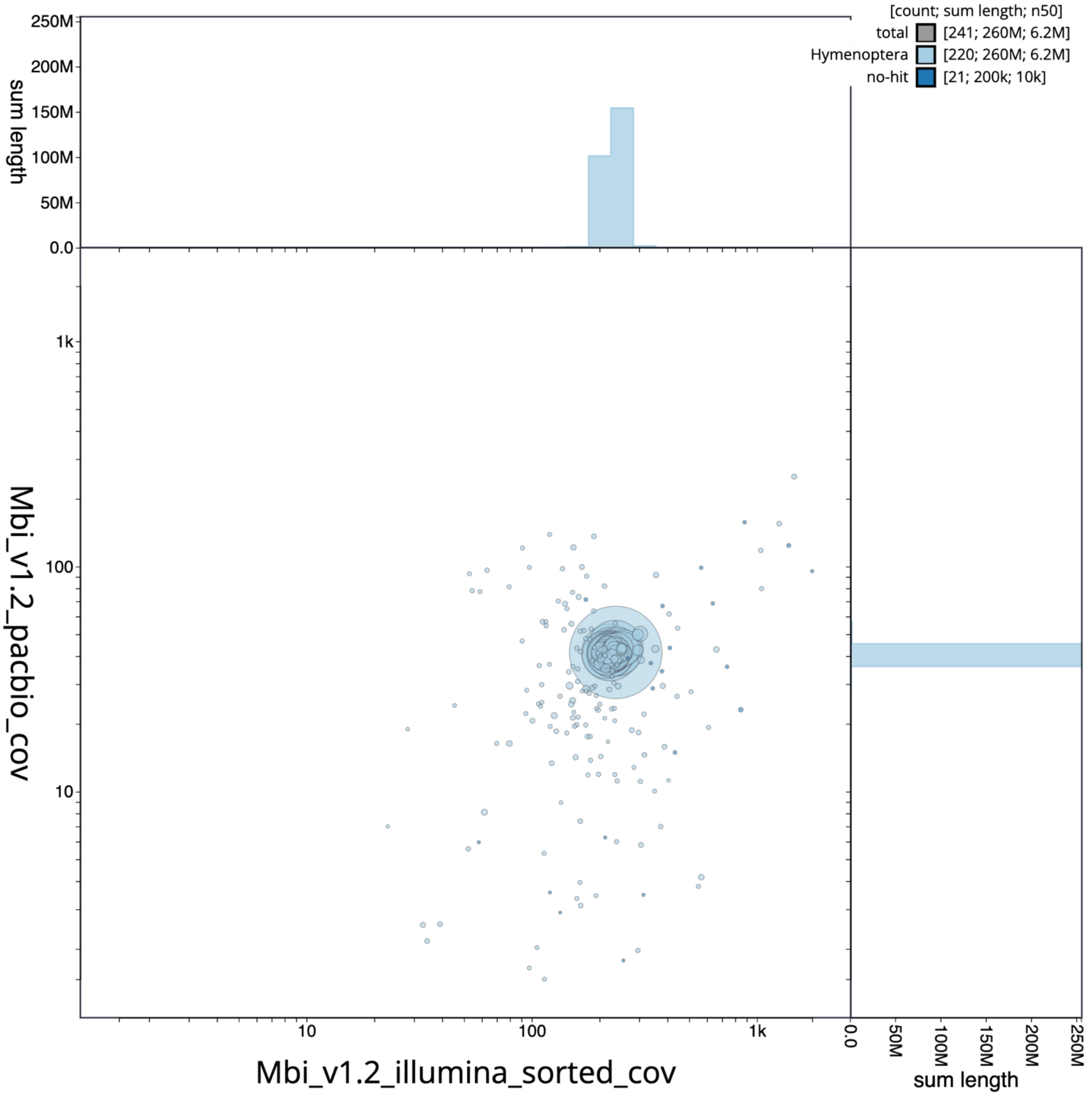

Figure S2 Sequencing coverage of short reads (x-axis) against long reads (y-axis) showing that coverage of the assembled scaffolds was mostly congruent between the two sequencing technologies as we removed outlier scaffolds (i.e., scaffolds with coverage <1x) during quality trimming steps. Circle size represents scaffold size.

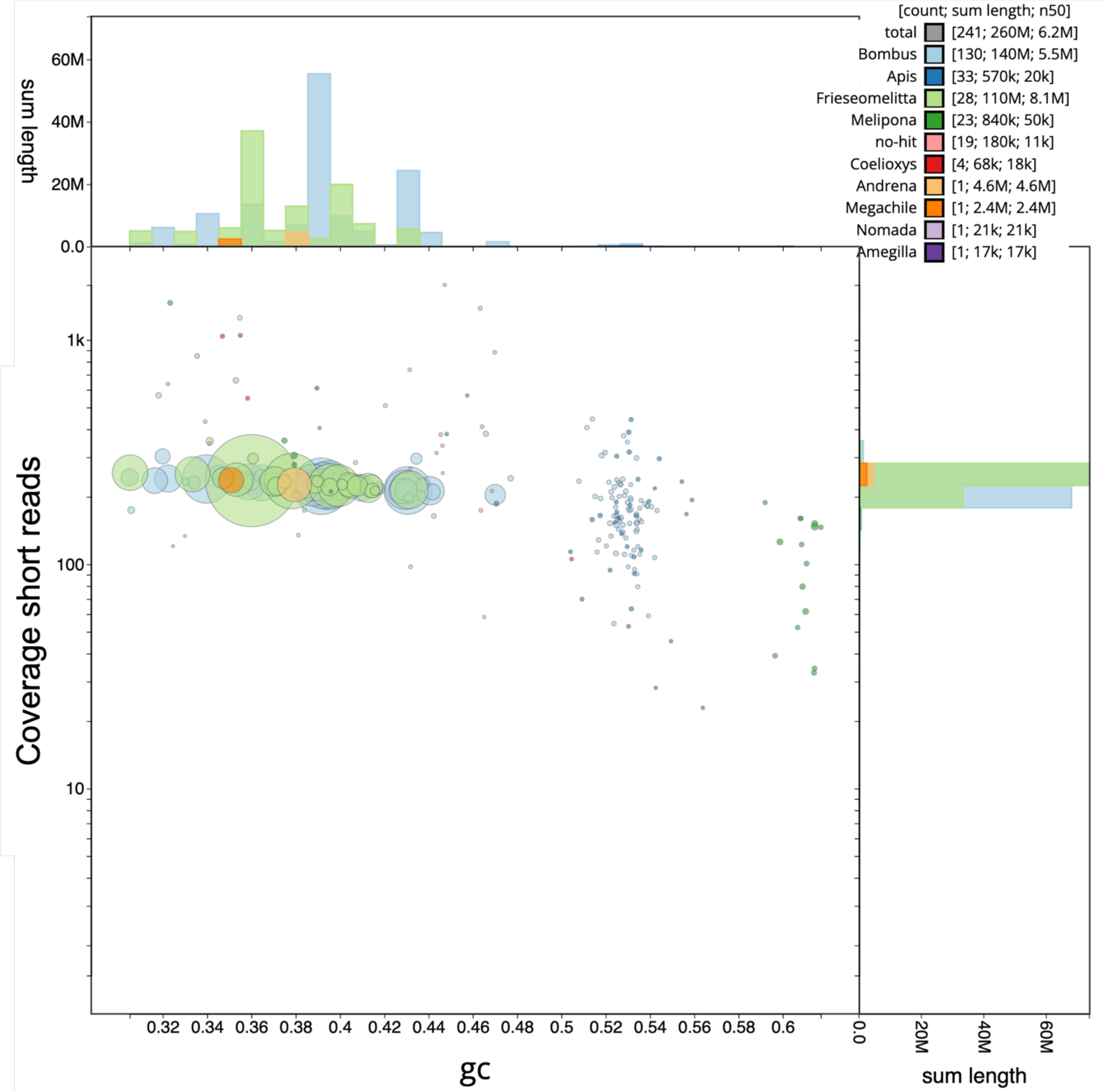

Figure S3 Blob plot of short reads base coverage against the GC proportion of scaffolds in the genome assembly of *Melipona bicolor*. Sequences are colored by matching genus sequences in databases. Circle size represents scaffold size. The histograms show the sequence length distribution in each axis.

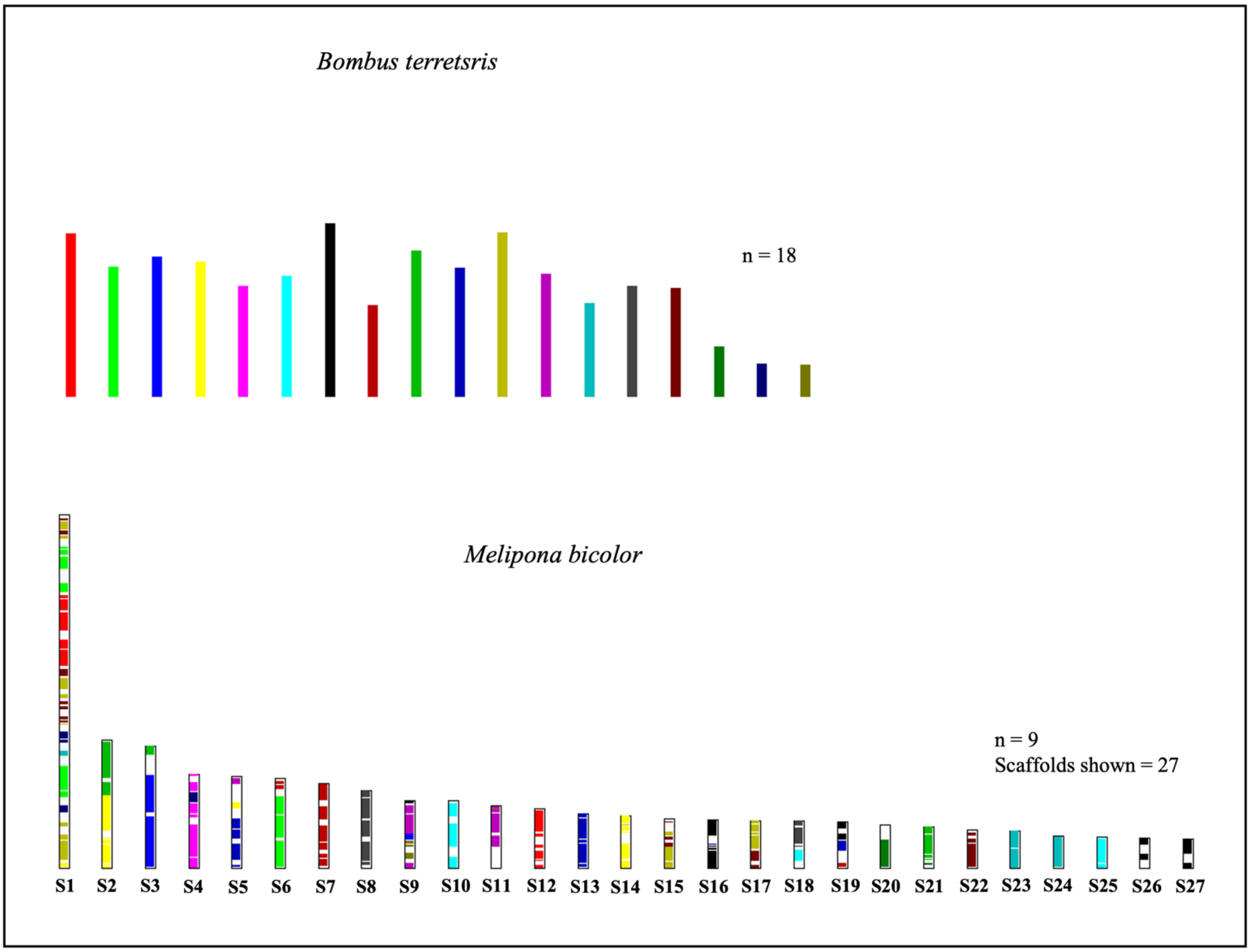

Figure S4 Genomic synteny inferred between the genome assembly of *B. terrestris* (upper bars), and *M. bicolor* (lower bars). The same colors represent syntenic chromosome regions in the *B. terrestris* genome. For *M. bicolor* the largest 27 scaffolds are presented instead of the chromosomes, these represent 80% of the total genome assembly.

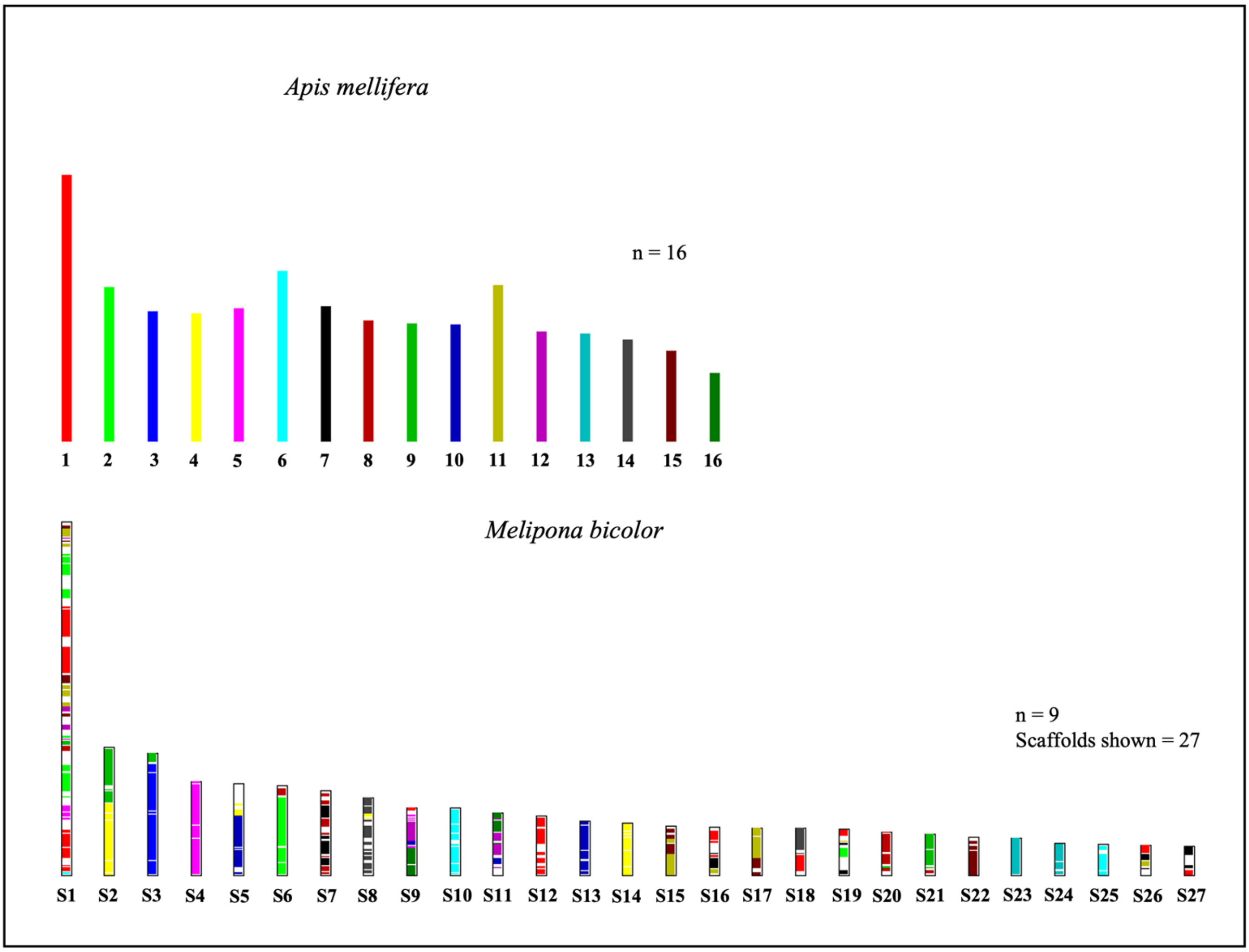

Figure S5 Genomic synteny inferred between the genome assembly of *A. mellifera* (upper bars), and *M. bicolor* (lower bars). The same colors represent syntenic chromosome regions in the *A. mellifera* genome. For *M. bicolor* the largest 27 scaffolds are presented instead of the chromosomes, these represent 80% of the total genome assembly.
